# Ganitumab and metformin plus standard neoadjuvant therapy in stage 2/3 breast cancer

**DOI:** 10.1038/s41523-021-00337-2

**Published:** 2021-10-05

**Authors:** Douglas Yee, Claudine Isaacs, Denise M. Wolf, Christina Yau, Paul Haluska, Karthik V. Giridhar, Andres Forero-Torres, A. Jo Chien, Anne M. Wallace, Lajos Pusztai, Kathy S. Albain, Erin D. Ellis, Heather Beckwith, Barbara B. Haley, Anthony D. Elias, Judy C. Boughey, Kathleen Kemmer, Rachel L. Yung, Paula R. Pohlmann, Debu Tripathy, Amy S. Clark, Hyo S. Han, Rita Nanda, Qamar J. Khan, Kristen K. Edmiston, Emanuel F. Petricoin, Erica Stringer-Reasor, Carla I. Falkson, Melanie Majure, Rita A. Mukhtar, Teresa L. Helsten, Stacy L. Moulder, Patricia A. Robinson, Julia D. Wulfkuhle, Lamorna Brown-Swigart, Meredith Buxton, Julia L. Clennell, Melissa Paoloni, Ashish Sanil, Scott Berry, Smita M. Asare, Amy Wilson, Gillian L. Hirst, Ruby Singhrao, Adam L. Asare, Jeffrey B. Matthews, Nola M. Hylton, Angela DeMichele, Michelle Melisko, Jane Perlmutter, Hope S. Rugo, W. Fraser Symmans, Laura J. van‘t Veer, Donald A. Berry, Laura J. Esserman

**Affiliations:** 1 Masonic Cancer Center, University of Minnesota, 420 Delaware St., SE, MMC 480, Minneapolis, MN 55455 USA; 2 Georgetown University, 3800 Reservoir Rd, NW, Washington, DC 20007 USA; 3 University of California San Francisco Department of Laboratory Medicine, 2340 Sutter Street, S433, San Francisco, CA 94115 USA; 4 Mayo Clinic Rochester c/o Merck Corporation, 126 E. Lincoln Ave Rahway, New Jersey, 07065 USA; 5 Mayo Clinic Division of Medical Oncology, 200 1st St SW, Rochester, MN 55905 USA; 6 University of Alabama at Birmingham c/o Seattle Genetics, 21823 30th Drive S.E., Bothell, WA 98021 USA; 7 University of California San Francisco Division of Hematology-Oncology, 550 16th Street, San Francisco, CA 94158 USA; 8 University of California San Diego Department of Surgery, 3855 Health Sciences Dr, M/C 0698, La Jolla, CA 92093 USA; 9 Yale University Medical Onciology, 111 Goose Lane, Fl 2, Guilford, CT 06437 USA; 10 Loyola University Chicago Stritch School of Medicine Cardinal Bernardin Cancer Center, 2160 South First Ave, Maywood, IL 60153 USA; 11 Swedish Cancer Institute Medical Oncology, 1221 Madison Street, Seattle, WA 98104 USA; 12 UT Southwestern Medical Center Division of Hematology-Oncology, 5323 Harry Hines Blvd, Bldg E6.222D, Dallas, TX 75390-9155 USA; 13 University of Colorado Anschutz Medical Center Division of Medical Oncology, 1665 Aurora Ct., Rm. 3200, MS F700, Aurora, CO 80045 USA; 14 OHSU Knight Cancer Institute South Waterfront Center for Health and Healing, 3303 SW Bond Ave Building 1, Suite 7, Portland, OR 97239 USA; 15 University of Washington Seattle Cancer Care Alliance, 825 Eastlake Ave East, Seattle, WA 98109-1023 USA; 16 MD Anderson Cancer Center, 1515 Holcombe, Houston, Texas 77030 USA; 17 University of Pennsylvania Division of Hematology-Oncology 3 Perelman Center, 3400 Civic Center Blvd, Philadelphia, PA 19104 USA; 18 Moffit Cancer Center, 2902 USF Magnolia Drive, Tampa, FL 33612 USA; 19 University of Chicago Section of Hematology/Oncology, 5841S. Maryland Avenue, MC 2115, Chicago, IL 60437 USA; 20 University of Kansas Division of Oncology, 2330 Shawnee Mission Pkwy, Ste 210, Westwood, KS 66205 USA; 21 Inova Medical Group, 3580 Joseph Siewick Dr 101, Fairfax, VA 22033-1764 USA; 22 George Mason University Institute for Advanced Biomedical Research, 10920 George Mason Circle Room 2008, MS1A9, Manassas, Virginia 20110 USA; 23 University of Alabama at Birmingham Hematology/Oncology, 1802 Sixth Avenue South 2510, Birmingham, AL 35294-3300 USA; 24 Wilmot Cancer Institute Pluta Cancer Center, 125 Red Creek Drive, Rochester, NY 14623 USA; 25 University of California San Francisco, 550 16th Street, 6464, San Francisco, CA 94158 USA; 26 University of California San Diego Division of Hematology-Oncology, 9400 Campus Point Dr, La Jolla, CA 92037 USA; 27 University of California San Francisco c/o Global Coalition for Adaptive Research, 1661 Massachusetts Ave, Lexington, MA 02420 USA; 28 University of California San Francisco c/o IQVIA, 135 Main St 21 floor, San Francisco, CA 94105 USA; 29 Point Eden Way, Hayward, CA 94545 USA; 30 Berry Consultants, LLC 3345 Bee Cave Rd Suite 201, Austin, TX 78746 USA; 31 Quantum Leap Healthcare Collaborative, 3450 California St, San Francisco, CA 94143 USA

**Keywords:** Breast cancer, Targeted therapies

## Abstract

I-SPY2 is an adaptively randomized phase 2 clinical trial evaluating novel agents in combination with standard-of-care paclitaxel followed by doxorubicin and cyclophosphamide in the neoadjuvant treatment of breast cancer. Ganitumab is a monoclonal antibody designed to bind and inhibit function of the type I insulin-like growth factor receptor (IGF-1R). Ganitumab was tested in combination with metformin and paclitaxel (PGM) followed by AC compared to standard-of-care alone. While pathologic complete response (pCR) rates were numerically higher in the PGM treatment arm for hormone receptor-negative, HER2-negative breast cancer (32% versus 21%), this small increase did not meet I-SPY’s prespecified threshold for graduation. PGM was associated with increased hyperglycemia and elevated hemoglobin A1c (HbA1c), despite the use of metformin in combination with ganitumab. We evaluated several putative predictive biomarkers of ganitumab response (e.g., IGF-1 ligand score, IGF-1R signature, IGFBP5 expression, baseline HbA1c). None were specific predictors of response to PGM, although several signatures were associated with pCR in both arms. Any further development of anti-IGF-1R therapy will require better control of anti-IGF-1R drug-induced hyperglycemia and the development of more predictive biomarkers.

## Introduction

The type I insulin-like growth factor receptor (IGF-1R) has been implicated in breast cancer growth, proliferation, and survival^1^. There are a number of approaches to disrupt IGF signaling, including receptor directed monoclonal antibodies (moAbs), tyrosine kinase inhibitors, and ligand neutralizing antibodies^2^. In breast cancer, clinical testing of these strategies has primarily focused on hormone receptor (HR)-positive cancers refractory to first-line endocrine agents. No studies to date have demonstrated a clinical benefit to adding an anti-IGF-1R inhibitor compared to endocrine therapy alone. A number of factors may contribute to the failure of this strategy, including lack of IGF-1R expression in endocrine-resistant breast cancer and induction of hyperglycemia and hyperinsulinemia caused by IGF-1R mAbs^2–4^. There is also evidence that the highly related insulin receptor may be a resistance pathway for anti-IGF-1R therapies, as it is expressed in many cancers^5^. Thus, inhibiting IGF-1R while controlling hyperinsulinemia may be an effective cancer therapy.

In breast cancer, the inhibition of IGF-1R in treatment naïve breast cancer alone or in combination with cytotoxic chemotherapy has not previously been evaluated. Previous preclinical work using IGF-1R mAbs argued that the inhibition of IGF-mediated survival pathways could enhance cytotoxic cell death^6^.

The monoclonal antibody ganitumab (AMG479) was developed to bind IGF-1R and has been tested in multiple settings. As a single agent, the phase 1 study of ganitumab demonstrated little toxicity and no MTD was reached^7^. In this study, the levels of serum ligand (IGF-1) were increased on drug exposure due to the disruption of the negative feedback pathway between growth hormone and IGF-1. Hyperglycemia was seen at the highest dose levels and patients without a diagnosis of diabetes occasionally required the institution of insulin or an oral hypoglycemic agent. In patients with known diabetes, ganitumab worsened glucose control. For the entire population, increases in hemoglobin A1C (HbA1c) were noted. Using a different IGF-1R mAb, figitumumab (CP-751,871) insulin levels during a phase 1 study showed consistent elevation^8^. These findings are explained by insulin resistance induced by elevated growth hormone levels and subsequent release of free-fatty acids from the liver^7^.

In this report, we evaluate a treatment regimen of paclitaxel, ganitumab, and metformin (PGM) compared to paclitaxel alone in HER2-negative operable breast cancer in the I-SPY2 trial. I-SPY2 is a neoadjuvant, adaptively randomized, multi-centre phase 2 platform trial evaluating investigational therapies in combination with standard-of-care chemotherapy for breast cancer at high risk of recurrence^9–11^. Pathologic complete response (pCR) is the primary endpoint. Metformin was added to the regimen since ganitumab-induced hyperglycemia was a known side effect of this therapy. Further, metformin alone has been reported to increase the pCR rate in the neoadjuvant therapy of breast cancer^12^. We also aimed to test the hypothesis that pre-treatment IGF-1R axis signaling at the expression level associates with response to IGF-1R inhibition, by examining eleven putative IGF-1R signaling axis biomarkers.

## Results

### Patient population

**Fig. 1:**
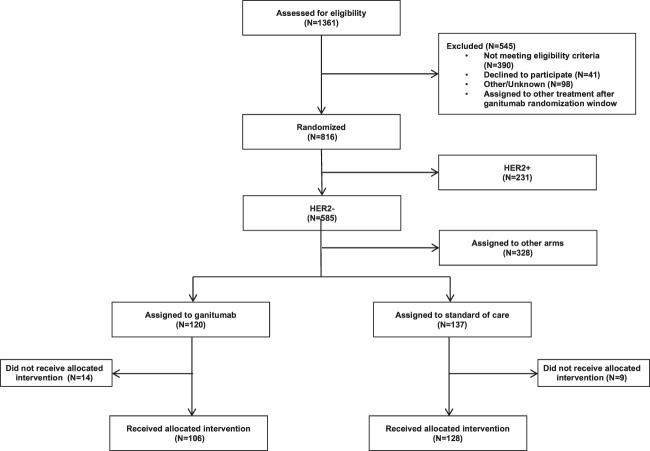
CONSORT diagram for paclitaxel, ganitumab, metformin arm in I-SPY2.

Between July 2012 and February 2015, 106 patients with HER2-negative tumors received paclitaxel/ganitumab/metformin (PGM) while 128 contemporary control patients (derived from the start of the trial in March 2010 through deactivation of the PGM arm in February 2015) received paclitaxel alone (Fig. 1). In the PGM arm, 14 patients did not receive assigned therapy and in the standard of care control arm, 9 patients did not receive allocated therapy and are not included in the analysis. Patients with HER2-positive tumors were not included in the PGM arm due to the lack of safety data for ganitumab in combination with trastuzumab. All patients received AC after completing the regimen. Baseline characteristics were similar between both arms, although there were slightly more participants with MammaPrint Hi2 status (56% versus 45%) in the experimental arm (Table 1).

**Table 1:** Baseline characteristics of participants in each arm.

Characteristic	Ganitumab (*n* = 106)	Control (*n* = 128)
Median age (range), yr	48 (23–70)	47.5 (24–77)
Ethnicity, *n* (%)		
White	86 (81%)	101 (79%)
African American	12 (11%)	18 (14%)
Asian	8 (8%)	7 (5%)
Other/Mixed	0 (0%)	2 (2%)
HR Status, *n* (%)		
Positive	58 (55%)	66 (52%)
Negative	48 (45%)	62 (48%)
Mammaprint Status, *n* (%)		
MP.Hi1	47 (44%)	70 (55%)
MP.Hi2	59 (56%)	58 (45%)
Median Tumor Size by MRI (range), cm	3.6 (0.8–14.7)	3.9 (1.2–15)
Baseline node status, *n* (%)		
Palpable	26 (25%)	60 (47%)
Non-palpable	67 (63%)	59 (46%)
N/A	13 (12%)	9 (7%)

### Efficacy

The estimated pCR rate was similar for the 106 HER2-negative patients enrolled on the PGM arm compared to those on the paclitaxel control arm (Table 2, Supplementary Fig. 1). In the HR-negative/HER2-negative subtype, pCR rates were higher in the PGM arm compared to control and had a 91% probability of being superior to control, though the arm did not meet I-SPY2’s prespecified threshold for graduation (≥85 probability of success in a hypothetical phase 3 controlled trial) in any of the subtypes (Table 2). For HR-positive/HER2-negative patients, the addition of ganitumab and metformin to paclitaxel showed no benefit over control. There was no evidence of improvement in event-free survival for the PGM arm at median follow-up of 4.1 years (Supplementary Fig. 2).

**Table 2:** Final predictive probabilities of success of ganitumab and metformin with paclitaxel followed by anthracyclines in HER2− biomarker signatures. The combination failed to graduate in any of the three signatures.

Biomarker signature	Estimated rate of pathologic complete response % (95% Probability Interval)		Prob. superior to control, %	Predictive prob. of success in phase III Trial, %
	Ganitumab *n* = 106	Control *n* = 128		
All HER2−	22 (13–31)	16 (10–23)	89	33
HR+/HER2−	14 (4–24)	12 (4–19)	61	21
HR−/HER2−	32 (17–46)	21 (11–32)	91	51

### Safety and toxicity

Ten (9.4%) patients in the PGM arm and 11 (8.9%) patients receiving control therapy had dose reductions (Table 3). Patients assigned to PGM had a 30.2% rate of early discontinuation, mostly due to toxicity. In contrast, 24.2% of standard of care patients discontinued therapy, 7.0% discontinuing because of toxicity.

**Table 3:** Grade 3–4 adverse events experienced by greater than 1% of participants in either the ganitumab + metformin or control arm.

Adverse event	Ganitumab *n* = 106	Control *N* = 128
Neutrophil count decreased	20 (18.9%)	13 (10.2%)
Febrile neutropenia	14 (13.2%)	9 (7.0%)
Hyperglycemia	9 (8.5%)	1 (0.8%)
Anemia	8 (7.5%)	8 (6.3%)
Vomiting	7 (6.6%)	0 (0.0%)
Hypertension	4 (3.8%)	4 (3.1%)
Alanine aminotransferase increased	4 (3.8%)	3 (2.3%)
Diarrhea	4 (3.8%)	3 (2.3%)
Bone pain	3 (2.8%)	3 (2.3%)
Lymphocyte count decreased	3 (2.8%)	3 (2.3%)
Syncope	3 (2.8%)	2 (1.6%)
Urinary tract infection	3 (2.8%)	0 (0.0%)
Stomatitis	2 (1.9%)	3 (2.3%)
Neutropenia	2 (1.9%)	2 (1.6%)
Back pain	2 (1.9%)	1 (0.8%)
Dehydration	2 (1.9%)	1 (0.8%)
Pneumonitis	2 (1.9%)	1 (0.8%)
Sepsis	2 (1.9%)	1 (0.8%)
Anaphylactic reaction	2 (1.9%)	0 (0.0%)
Dyspnea	2 (1.9%)	0 (0.0%)
Nausea	2 (1.9%)	0 (0.0%)
Pulmonary embolism	2 (1.9%)	0 (0.0%)
Hypokalemia	1 (0.9%)	4 (3.1%)
Hyponatremia	1 (0.9%)	2 (1.6%)
Premature menopause	1 (0.9%)	2 (1.6%)
Peripheral sensory neuropathy	0 (0.0%)	2 (1.6%)
Dose reductions, *n* (%)	10 (9.4%)	11 (8.9%)
Early discontinuation, *n* (%)		
All reasons	32 (30.2%)	31 (24.2%)
Toxicity	29 (18.9%)	9 (7.0%)
Progression	8 (7.5%)	10 (7.8%)
Other	4 (3.8%)	12 (9.4%)
Median time to surgery, days (range)	168 (64–313)	165 (100–289)

Adverse events experienced in the PGM arm differed from standard of care therapy in several areas. Participants in the PGM arm showed increased grade 3/4 nausea (1.9% vs. 0%) and vomiting (6.6% vs. 0%), both of which are known metformin toxicities (Table 3). Infections were higher (9.4% versus 3.9%) in the experimental arm, as were reports of grade 3/4 neutropenia (18.9% versus 10.2%) in the experimental arm.

Despite the addition of metformin, hyperglycemia was common in patients receiving ganitumab. Hyperglycemia (all grades) was observed in 19.8% of PGM treated patients compared to 2.4% of control patients. Grade 3/4 hyperglycemia was seen in 8.5% of patients receiving PGM compared to 0.8% in the control arm.

To further evaluate glucose control, HbA1c was measured before, during and after therapy. Of the 106 patients assigned to the PGM arm, 105 had at least one measurement of HbA1c and 80 of these patients had more than one measurement during the trial.

The median baseline value of HbA1c was 5.4% (*n* = 104); 27% (*n* = 28) had baseline values greater than 5.7, the upper limit of normal as defined by the National Institute of Diabetes and Digestive and Kidney Diseases. Further, 4 patients had HbA1c greater than 6.5% and met this criterion for diabetes. Thus, a significant number of patients had abnormalities in HbA1c consistent with insulin resistance at baseline. No patients were taking any anti-diabetic therapies at the time of entry to the study.

**Fig. 2:**
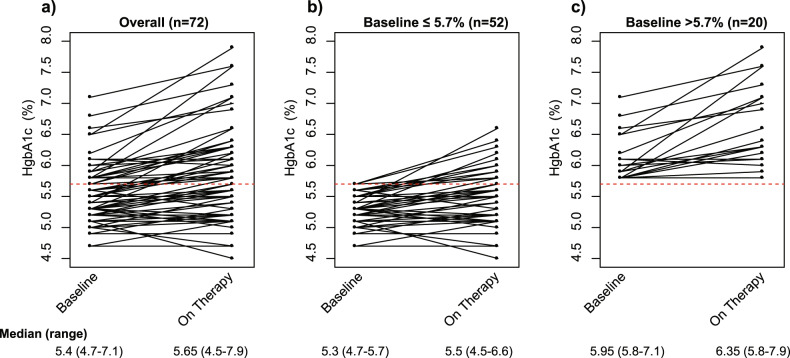
Changes in HbA1c at baseline and subsequent levels measured while on PGM therapy for patients with more than one measurement. (**a**) all patients (*n* = 72); (**b**) those with normal baseline HbA1c (*n* = 52); (**c**) elevated baseline HbA1c (*n* = 20).

To evaluate the effect of ganitumab/metformin on baseline HbA1c, we studied the 72 patients who had baseline HbA1c measurements and at least one additional measurement while on PGM therapy measured on average at 56 days (sd 16 days) after starting PGM. Figure 2a shows the changes in HbA1c at baseline and subsequent levels measured while on PGM therapy for all patients with more than one measurement; this overall population is then sub-divided into those with normal baseline levels (<5.7%) (Fig. 2b) and elevated levels at baseline (≥5.7%) (Fig. 2c). For patients with normal levels at baseline, 14 of 52 (27%) had a subsequent elevation of HbA1c above 5.7% while on therapy (Fig. 2b). HbA1c levels while on PGM were consistent, independent of the duration of PGM therapy. For those patients whose baseline HbA1c was already elevated, all 20 patients maintained elevated levels (Fig. 2c). These data are consistent with previous observations regarding the effect of ganitumab on exacerbating insulin resistance in both patients with normal and elevated baseline HbA1c. Further, for most patients, metformin administration did not seem to control glucose levels and this was particularly notable in the patients who had baseline elevation in HbA1c.

### Assessment of biomarkers predictive of pCR

**Fig. 3:**
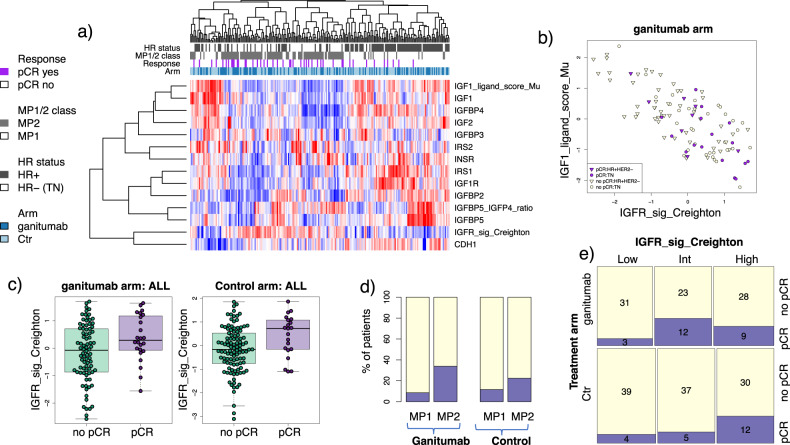
Candidate expression biomarkers in the IGF-1R pathway. **a** Heatmap of biomarkers evaluated. Patients are along columns and biomarkers are along the rows. Red indicates higher expression and blue lower. Annotation tracks reflects response (purple: pCR), HR status (black: HR+), MP 1/2 class (gray: MP2), and arm (dark blue: ganitumab). **b** Scatter plot showing IGF-1-ligand score vs IGFR Signature, (**c**) boxplots showing IGFR Signature levels in pCR vs. non-pCR patients in each arm (center line is group median; upper and lower limits of the box correspond to the 1st and 3rd quartile with whiskers extending to 1.5 times the interquartile range from top/bottom of the box); **d**–**e** pCR rate in (**d**) the MP1 vs. MP2 class and (**e**) IGFR-Signature groups by arm.

Expression levels of eleven putative IGF-1R signaling axis biomarkers are shown in the heatmap in Fig. 3a. The IGF-1 ligand signature^13^ strongly correlates with IGF-1 and IGF-2 expression, as well as IGFBP4, while the IGF-1R signature^14^ shows a strong inverse correlation (Pearson *r* = −0.79) (Fig. 3a, b). In the population as a whole, lower levels of IRS1 and IGFBP5 significantly associated with better response to PGM (LR *p* < 0.05), as did lower levels of the signature IGF-1 ligand score and higher levels of the IGF-1R signature (Fig. 3c; Supplementary Table 1). However, levels of IRS1 and the two expression signatures also trend toward or are significantly associated with response in the control arm, and treatment interactions for all four biomarkers are non-significant (LR *p* > 0.05). Therefore, these signatures do not qualify as specific predictors of response to PGM per our definition. High MammaPrint scores (MP2) were also associated with higher pCR rates in both PGM and Control arms (Fig. 3d).

As the Creighton IGF-1R signature was previously evaluated in tertiles (low, intermediate, high)^14^, we also assessed this signature as a categorical variable. Similar to the continuous case, IGF-1Rsig-class associates with pCR in both the PGM and control arms (Fisher test two-sided *p* = 0.033 and 0.044, respectively; Fig. 3e).

In receptor subset analysis, low levels of IGFBP5 and IGF2 trended toward association in the HR-negative/HER2-negative subset but did not reach significance (LR 0.05 < *p* < 1). There were no significant associations in the HR-positive/HER2-negative subset.

Finally, pCR rates are similar between patients with baseline HgbA1c ≤ 5.7% (21%) vs. >5.7% (25%) (Fisher test two-sided *p* = 0.79) in the PGM arm.

## Discussion

A wealth of evidence suggests an important role for IGF-1R signaling in many cancers. Lung, colon, prostate, glioblastoma, melanoma and breast, are known to exhibit increased expression of IGF-IR or its ligands, including evidence of signaling in primary breast cancer specimens^15,16^. A number of therapeutic agents, such as small molecules and monoclonal antibodies have been developed that target this complex pathway^17^. Initial phase 1 monotherapy trials targeting IGF-1R reported exceptional responses and prolonged disease control, but subsequent randomized phase 2 or phase 3 trials proved disappointing^17^.

Although multiple clinical trials have evaluated IGF-1R inhibitors in endocrine-resistant metastatic breast cancer, there is considerably less experience with agents targeting this pathway in operable breast cancer. Here, we combined ganitumab with conventional chemotherapy in the setting of neoadjuvant treatment of locally advanced, stage II/III breast cancer.

In the I-SPY2 trial, the combination of ganitumab, metformin and paclitaxel failed to graduate. Although the combination resulted in a modest increase in the pCR rate in triple-negative breast cancers, it did not meet the predefined thresholds for subsequent development. Molecular profiling studies have shown that TNBC is not a single entity. Lehman et al. identified four distinct subtypes with distinct biological differences^18^. While our study did not include enough TNBC patients to evaluate responses by molecularly defined subtypes, perhaps a subset of TNBC is sensitive to IGF-1R inhibition but this could not be distinguished in our trial.

While it has been suggested that serum IGF ligand levels may predict outcome to IGF-1R inhibitors^19^, no tumor-derived biomarkers have yet to be shown to predict benefit from this class of drugs. In this study, we examined the expression of several genes involved in the IGF signaling pathway for their association with response. Although sample sizes are relatively small, we observed no indication that pre-treatment expression levels of IGF-1R axis genes and signatures are specifically predictive of response to IGF-1R-targeted agents; this was also true in the triple-negative (HR-negative, HER2-negative) subset, in which there appeared to be a small treatment effect, with the pCR rate in PGM higher than that of controls (33% vs 21%). The inverse correlation between the IGF-ligand signature and IGF-1R activation signature has previously been noted^19^. While this may seem counterintuitive, the IGF-1 ligand signature was derived from tissues expressing high levels of the ligand along with other associated genes such as the IGF binding proteins. The IGF ligand system is complex and levels of “available” ligand are regulated by IGF binding proteins, these binding proteins prevent ligand receptor interactions. It has been argued that the IGF-I ligand signature represents more differentiated tumors and these tumors may not be driven by IGF-1R signaling as reflected in the IGF-1R activation signature. These results suggest either that pre-treatment expression levels/signatures cannot adequately capture IGF-1R signaling dynamics, or that PGM was unable to suppress signaling of this pathway as suggested by the HbA1c data, or that PGM functions in an off-target manner.

As it is well known that IGF-1R-blocking agents can cause insulin resistance, hyperinsulinemia and hyperglycemia as a class effect^20^, metformin was administered concomitantly with ganitumab and paclitaxel. Metformin was selected due to its safety record and long use in patients with type 2 diabetes^21^. Further, metformin by itself has been associated with improved pCR rates in diabetic patients receiving neoadjuvant therapy^12^. In I-SPY2, HbA1c levels demonstrated that blood glucose levels were not uniformly controlled with metformin, as 29% of the patients on the PGM arm exceeded the clinical threshold for hyperglycemia of HbA1c >5.7%. Further, 30% of patients with normal HbA1c at study entry subsequently had elevated levels after receiving PGM. While the exact cause of these changes in glucose regulation could be multifactorial since patients were also receiving dexamethasone during paclitaxel treatment, the results are consistent with the known effects of IGF-1R mAbs^2^. Further, dietary strategies (ketogenic diet) or drugs (SGLT2 inhibitors might be more effective in controlling hyperglycemia as has been suggested in studies of PI3K inhibitors^22,23^.

Induction of hyperglycemia results in elevated levels of insulin. While we did not measure serum levels of insulin in this trial, all of the prior trials that measured insulin levels showed that IGF-1R inhibitors increased insulin^8^. Insulin receptor is a known signaling pathway in breast cancer and it has been suggested that insulin receptor activation results in a kinome signature distinct from IGF-1R and also associated with poor outcome^24^. In a previous trial testing the IGF-1R antibody figitumumab in combination with endocrine therapy for front line treatment of metastatic disease, patients with normal glucose control as defined by HbA1c less than 5.7% had a trend toward benefit with the addition of figitumumab^25^.

Since inhibition of IGF-1R signaling enhances apoptotic responses to cytotoxic chemotherapy^6^, IGF-1R antibodies have been tested in combination with other chemotherapies. The first such report was in advanced non-small cell lung cancer with cytotoxic chemotherapy in combination with figitumumab. While the first report of a randomized phase 2 study was reportedly positive, these data were eventually withdrawn due to irregularities in response reporting^26^. The amended response rates still demonstrated a small benefit for figitumumab, but the primary endpoint of improvement in the overall response rate was not met. Ganitumab has been tested with gemcitabine in the therapy of metastatic pancreatic cancer. A randomized phase II study suggested that higher levels of circulating IGF ligands correlated with ganitumab benefit^19^. However, a larger phase III trial were unable to show additional benefit beyond gemcitabine by the addition of ganitumab. Further, the original promising biomarker data could not be reproduced in this trial. Thus, these data show that the promising preclinical data could not be reproduced in clinical trials and it seems likely that the disruption of glucose homeostasis by this class of drugs could be a reason for this failure^4^.

In summary, ganitumab and metformin did not graduate from I-SPY2 despite a small increase in pCR rates in TN breast cancer. If IGF-1R inhibition has a role in combination with chemotherapy, then additional biomarkers are needed, likely including early-treatment biomarker response, to identify IGF-1R driven tumors. Further, drugs that do not increase hyperglycemia or hyperinsulinemia need to be developed to comprehensively target the ligands and receptors in this complex system.

## Methods

### Study design

I-SPY2 is an ongoing, open-label, adaptive, randomized phase II multicenter trial of NAT for early-stage breast cancer at high risk of recurrence (clinicaltrials.gov/NCT01042379). It is a platform trial evaluating multiple investigational arms in parallel, each consisting of standard neoadjuvant therapy plus with an investigational agent compared to a shared standard of care control arm. The primary endpoint is pathological complete response (pCR) after completion of all chemotherapy, defined as the absence of invasive tumor in breast and regional nodes (ypT0/is and ypN0). In the event a participant switches to a non-protocol assigned therapy, forgoes surgery, or withdraws from the trial, they are considered “non-pCR” during analysis. Secondary endpoints include residual cancer burden (RCB), 3-year event-free survival (EFS) and distant relapse-free survival (DRFS). All patients are followed for long-term outcome.

Clinical biomarker assessments are determined at baseline to group patients into one of 8 subtypes based on hormone receptor (HR) status, HER2-receptor, and 70-gene assay results (MammaPrint^®^ (MP) Agendia). MP scores were divided into Hi1 or Hi2 (<−0.573) based on previous data from the I-SPY trial^27^. Adaptive randomization in I-SPY2 preferentially assigns patients to agents according to actively updated Bayesian posterior probabilities of pCR rate within each subtype; 20% of patients are randomized to control.

Agents ‘graduate’ from I-SPY2 by reaching, in any of 10 clinically relevant signatures, a predefined efficacy threshold of 85% probability of success in a signature-specific, hypothetical 300-patient, 1:1 confirmatory phase 3 trial. Agents may be dropped for futility if the predicted probability <10% for all signatures or the maximum enrollment threshold has been reached for that arm. Additional details on the study design have been published previously^28,29^.

### Eligibility

Patients eligible for I-SPY2 are women ≥18 years, with stage II or III breast cancer and primary tumors >2.5 cm by clinical exam or >2.0 cm by imaging, with Eastern Cooperative Oncology Group performance status of 0 or 1. MammaPrint low-risk HR-positive/HER2-negative patients are excluded from I-SPY2 as their lower risk of recurrence does not justify risks of exposure to an investigational agent^30^. All participants provide written informed consented prior to screening and again prior to treatment. Only HER2-negative patients were eligible for randomization to the PGM.

### Treatment

Participants in the control arm received 80 mg/m^2^ intravenous paclitaxel weekly for 12 weeks; those in the ganitumab arm also received ganitumab 12 mg/kg iv q2 weeks, and 850 mg metformin PO BID concurrently with paclitaxel. All participants then received four cycles of chemotherapy consisting of 60 mg/m^2^ doxorubicin plus 600 mg/m^2^ cyclophosphamide iv every 2–3 weeks (AC).

Definitive surgery followed AC, with lumpectomy or mastectomy at the discretion of the treating surgeon. Sentinel node dissection was allowed in node-negative patients, with axillary node dissection in node-positive patients according to NCCN and local practice guidelines^31^. Adjuvant treatment was not mandated by the trial, but was at the discretion of the treating oncologist. However, standard-of-care adjuvant therapy per NCCN guidelines was recommended.

### Assessments

Core biopsies and breast MRI were performed at baseline and following 3 weeks of therapy. Additional MRIs were performed between paclitaxel and AC and again following AC, as previously described^28,29^. Surgical specimens were analyzed for the primary endpoint by local pathologists trained in the residual cancer burden (RCB) method^32^.

### Trial oversight

The trial was designed by the I-SPY2 study investigators. Amgen provided study drug but played no role in the study design, collection/analysis of data or in manuscript preparation. All participating sites received local institutional review board approval. A DSMB meets monthly to review patient safety and study progress. The authors of the manuscript vouch for the accuracy and completeness of the data reported.

### Expression biomarkers analyzed

RNA extracted from patients’ fresh frozen pre-treatment biopsies were profiled using Agilent 44 K expression arrays (Agendia, Inc). To determine if IGF-1R signaling was associated with response to ganitumab, we evaluated the expression of 11 genes involved in IGF1R pathway signaling (IGF1, IGF2, IGF1R, INSR, IGFBP2, IRS1, IRS2, IGFBP3, IGFBP4, IGFBP5, CDH1), the IGFBP5/IGFBP4 ratio, and two IGFR expression signatures - IGF1_ligand_score_Mu^13^ and IGFR_sig_Creighton^14^, evaluated as previously published.

Briefly, IGF1_ligand_score_Mu^13^ scores were calculated as published by (1) reading in the signature gene list (274 genes); (2) extracting (centered) expression data for all available signature genes from the 233 patients in our study; (3) calculating pearson correlations and associated p-values for each signature gene and IGF1 across the population; (4) creating a ternery ‘sign’ vector from (3) with each entry equal to (−1, 0, or 1) for each signature gene based on the correlation results (−1 if negatively correlated, +1 if positively correlated, and 0 if *p* > 0.01); 5) calculating the Pearson correlation coefficient for the ternery vector from (4) and the signature genes for each patient. Correlation coefficients from (5) constitute the IGF1_ligand_score_Mu scores for each patient.

The IGFR_sig_Creighton signature^14^ was calculated as published by (1) reading in the signature gene list (802 genes), (2) median centering the data over the population, (3) creating a binary ‘sign’ vector (−1, 1) associated with the signature genes as published (+1: *n* = 364; −1: *n* = 438), and (4) calculating the t-statistic for the regression model x~sign_vector and multiplying by (−1). After z-scoring (mean = 0; sd = 1), the values from (4) constitute the IGFR_sig_Creighton scores for each patient.

We also measured glycosylated hemoglobin (HbA1c) as a measure of glucose control and potential biomarker of response.

### Statistical analysis

In the standard I-SPY2 Bayesian approach, probability distributions of pCR rate for each regimen in each subtype are updated continuously via a covariate analysis with HR, HER2 and MP status as covariates, adjusting for time trends to allow comparisons against I-SPY2 controls enrolled since the opening of I-SPY2. Adaptive randomization probabilities and the Bayesian probability that each regimen is superior to control are derived from these distributions. “Graduation” of a treatment arm occurs if the predicted pCR rate in any signature meets the pre-specified threshold of 85% probability of success in a hypothetical 300-patient, 1:1 randomized, phase 3 trial.

#### Contemporary controls and adjusting for time trends

The initial statistical analyses in I-SPY 2 compared investigational arms with concurrently randomized controls. The approach applied to the first five investigational arms: neratinib, veliparib+carboplatin, trebananib, ganitumab, and Akt inhibitor MK2206. In September 2013 the FDA granted accelerated approval for pertuzumab+trastuzumab+docetaxel as neoadjuvant therapy for high risk HER2+breast cancer. Our investigators and DSMB required dropping the I-SPY 2 control arm for HER2+ subtypes because it did not contain pertuzumab, which we did by amendment in early 2014. At the time pertuzumab+trastuzumab+paclitaxel (for the first 12 weeks of neoadjuvant therapy) was an investigational arm in the trial, but it had accrued only 6 patients with none through surgery.

We wanted to be able to use the results for the original control arm but were concerned about the possibility of a drift in the prognosis of patient population over time and within patient subtype. We built a model that we call “the time machine” that adjusts for the results over time within each arm, including result for the investigational arms as well as those for control. Having multiple arms in the trial with different time periods during which they are accruing patients enabled bridging across the different eras of trial accrual. The time machine discounts result from the past, with more discounting if they are further in the past. The mathematical basis and motivation was a statistical model for bridging eras in sports^33^. The model description follows.

The control rate for an investigational arm is adjusted to the time period when the arm was being randomized to patients. Each investigational arm is compared directly against its concurrently randomized controls. The time machine strengthens this comparison by bridging to earlier controls via a series of direct comparisons. These direct comparisons are the various comparisons of arms that have been randomized in the trial, including comparisons of investigational arms against each other as well as against controls. The strength of this borrowing depends on the time-period overlaps among the various arms, both control and investigational arms. The greater uncertainty associated with results during periods of relatively low accrual and when fewer arms are being randomized is incorporated into the final analyses of the various arms.

We explicitly incorporate terms in the model to account for potential time trends in the pCR rate; we account for molecular subtype and treatment as well. This is accomplished using time-dependent offset terms in a logistic model. Time is set to 0 at each analysis. We partition time in the past into bins of 90 days each. The index of the most recent bin, that for the previous 0–90 days, is 1. The index of the bin 91–180 days in the past is 2. And so on. Let *t*_*i*_ be the index of the bin for the randomization time of patient *i*.

We model time-trend parameters *δ*(*t*) within each bin *t*. These are additive parameters in the model for the log-odds ratio of pCR rate for each investigational arm compared with control. We use two sets of time-trend parameters, *δ*_+_(*t*) for HER2-positive and *δ*_–_(*t*) for HER2-negative. Consider patient *i* who has subtype (HR–, HER2+, MP–) and was randomized 750 days before present. Her bin *t*_*i*_ is 9 and her time-trend offset is *δ*_+_(*9*).

Suppressing subscripts + and – for both HER2 + and HER2–, we set *δ* (*t*) = 0 for *t* = 1, 2, 3, 4. That means the previous year’s results count fully in the analysis. Further in the past, that is, for *t* > 4, {*δ*(*t*)} is a second-order Normal Dynamic Linear Model (NDLM)^34^. The NDLM uses the data within bins to estimate the respective log-odds ratios, but it also serves to smooth the effect across bins.

The time machine has the following structure for both HER2+ and HER2–, again suppressing the + and – subscripts:

$$\delta \left( 1 \right) = \delta \left( 2 \right) = \ldots = \delta \left( 4 \right) = 0$$

$$\delta \left( 5 \right)\sim N\left( {\mu _0,\tau _0^2} \right)$$

$$\delta \left( 6 \right) - \delta \left( 5 \right)\sim N\left( {\mu _1,\tau _1^2} \right)$$

$$\delta \left( t \right) - 2\delta \left( {t - 1} \right) + \delta \left( {t - 2} \right)\sim N\left( {0,\tau ^2} \right)for\,{\it{t}} \,>\, 6$$

$$\tau ^2\sim IG\left( {\alpha ,\beta } \right)$$

In this notation, *N*(*μ*, *σ*^2^) refers to a normal distribution with mean *μ* and standard deviation *σ* and *IG* stands for inverse gamma. The parameters of the prior distributions are *μ*_*0*_ = *μ*_*1*_ = 0, $\tau _0^2$ = $\tau _1^2$ = 0.001, *α* = 1, *and β* = 0.001.

### Biomarker Analysis

In the predictive marker analysis, we employed a 3-step Qualifying Biomarker Evaluation method. In the predictive marker analysis, first we used logistic regression modeling to evaluate the relative performance of the marker within the experimental and control arms (models M1 and M2):

- M1 (PGM arm): pCR ~ marker
- M2 (Ctr arm): pCR ~ marker
  Second, we performed marker-treatment interaction testing, also using logistic regression modeling (model M3):
- M3: pCR ~ marker + Tx + (marker x Tx)

A biomarker was considered a specific predictor of response for PGM if it associated with response in that arm, and if the biomarker x treatment interaction was also significant (likelihood ratio (LR) test, p < 0.05). Analysis was also performed adjusting for HR status as a covariate (models M1-2b: pCR ~ marker + HR; and model M3b: pCR ~ marker + HR + Tx + (marker x Tx)), and numbers permitting, within receptor subsets. Biomarkers were assessed individually without adjustment for multiple hypothesis testing. Our statistics are descriptive rather than inferential and do not adjust for multiplicities. All analyses were performed in the computing environment R (v.3.6.3) in RStudio (v.1.2.5033), using R Packages ‘stats’ (v.3.6.3) and ‘lmtest’ (v.0.9-37).

### Reporting summary

Further information on research design is available in the Nature Research Reporting Summary linked to this article.

## Supplementary information

Supplementary information

Supplementary Data 1

Reporting Summary

## Data Availability

Clinical datasets that support Fig. 1 and Supplementary Figs. 1 and 2 are available upon request by email to ispyadmin@ucsf.edu. Biomarker data supporting Figs. 2 and 3 have been provided as a supplementary file. Patient-level expression data is available at https://www.ncbi.nlm.nih.gov/geo/query/acc.cgi?acc=GSE180962.

## Code availability

Biomarker data analysis was performed using R version 3.4.3 and Bioconductor; code available upon request from ispyadmin@ucsf.edu. The randomization engine and Bayesian analytic software used in efficacy analysis are used under license from Berry Consultants, LLC; requests for code should be directed to don@berryconsultants.com.
