# Supplementary material for: Ganitumab and metformin plus standard neoadjuvant therapy in stage 2/3 breast cancer
Source: NPJ Breast Cancer. 2021 Oct 5;7:131. doi: 10.1038/s41523-021-00337-2 (PMC8492731; doi:10.1038/s41523-021-00337-2)
Supplement: Supplementary file 1 — Supplementary information [file 41523_2021_337_MOESM1_ESM.pdf]

Supplementary Materials

Ganitumab and Metformin plus Standard Neoadjuvant Therapy in Stage 2/3 Breast Cancer: Results from the I-SPY2 Adaptively Randomized Platform Trial

Yee D, et al.

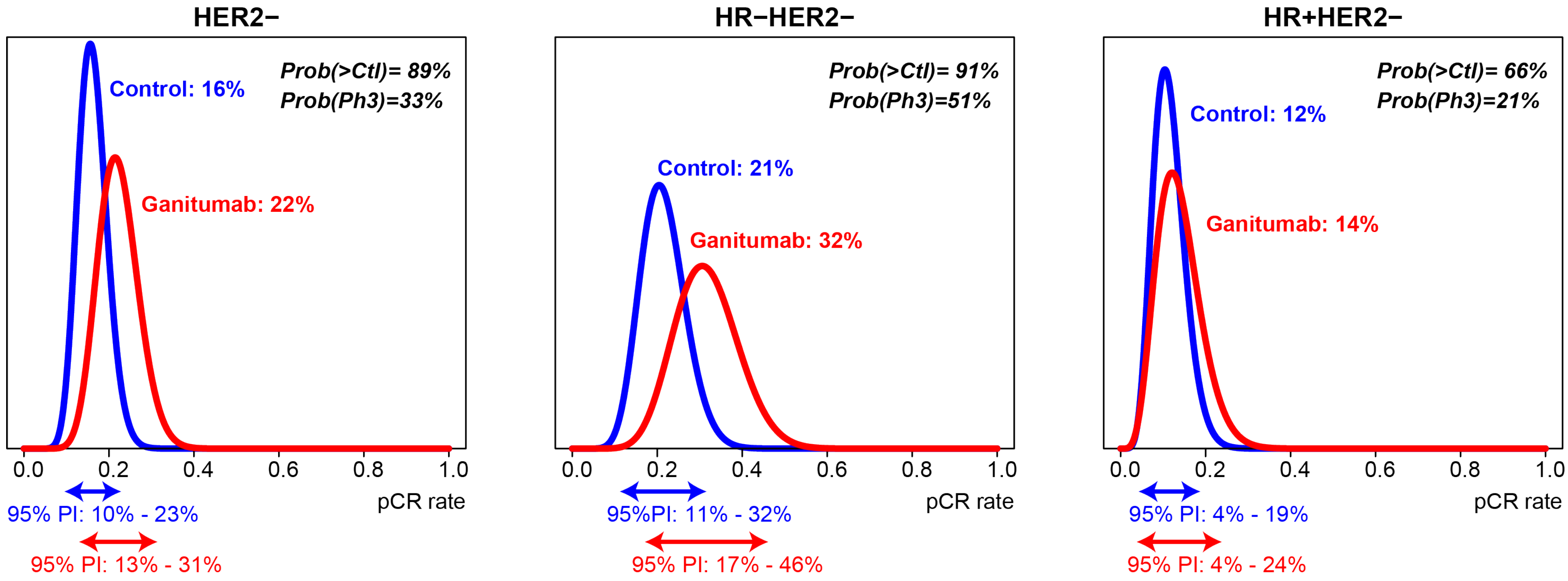

Supplementary Figure 1: Final posterior probability distributions of experimental and control arms for each HER2- biomarker signature

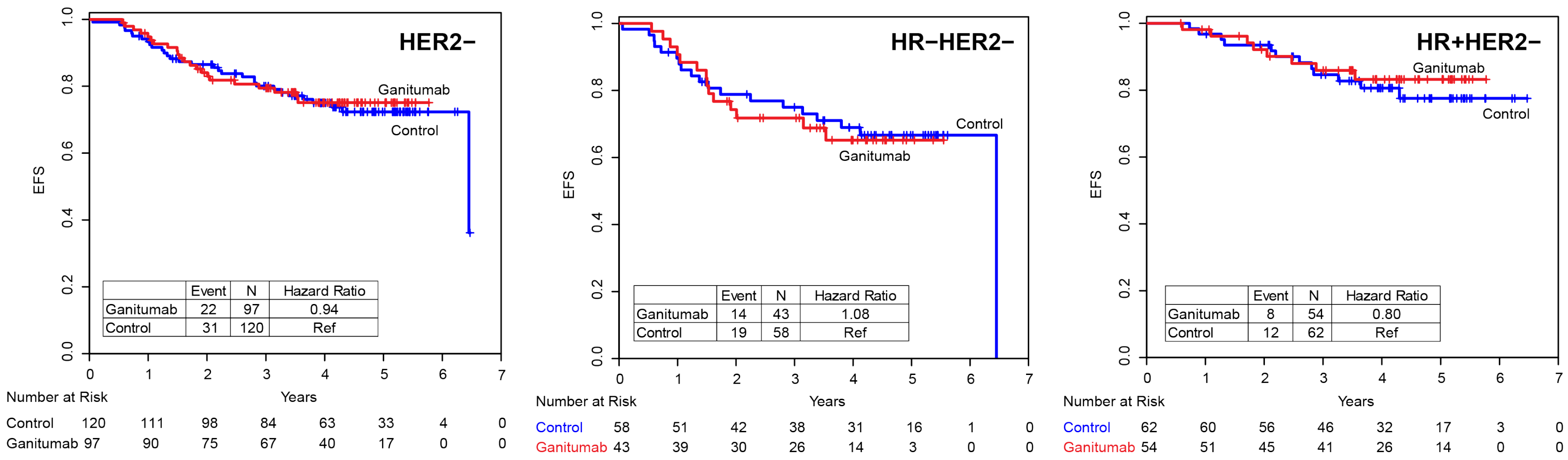

Supplementary Figure 2: Survival plots of patients in the ganitumab and control arms for all HER2- patients, HR-HER2- patients and HR+HER2- patients.

Supplementary Table 1

Gene/ signature	All subtypes						TN subset						HR+HER2- subset					
	Ganitumab (n=106)		Ctr (n=127)		All (n=233)		Ganitumab (n=48)		Ctr (n=62)		All (n=110)		Ganitumab (n=58)		Ctr (n=65)		All (n=123)	
	OR/unit inc	LR p	LR p (adj HR)	OR/unit inc	LR p	LR p (adj HR	Biomarker x treatment int	Biomarker x treatment int *	OR/unit inc	LR p	OR/unit inc	LR p	Biomarker x treatment int	OR/unit inc	LR p	OR/unit inc	LR p	Biomarker x treatment int
IGF1	0.946	0.83	0.882	0.759	0.242	0.266	0.535	0.553	0.931	0.833	0.877	0.629	0.891	1.01	0.977	0.568	0.195	0.354
IGF2	0.585	0.0564	0.128	0.694	0.191	0.27	0.687	0.694	0.555	0.0848	0.842	0.563	0.407	0.937	0.897	0.555	0.243	0.466
IGF1R	0.779	0.326	0.948	0.599	**0.0489**	0.133	0.488	0.57	0.503	0.11	0.76	0.538	0.514	1.71	0.162	0.522	0.136	**0.0412**
INSR	0.694	0.118	0.161	0.865	0.567	0.63	0.529	0.509	0.714	0.307	0.807	0.487	0.79	0.704	0.335	1.07	0.874	0.467
IGFBP2	0.787	0.344	0.931	0.66	0.0675	0.156	0.606	0.535	1.2	0.607	1.14	0.681	0.914	0.773	0.586	0.381	**0.00894**	0.24
IRS1	0.614	**0.0479**	0.304	0.619	0.0554	0.131	0.984	0.983	0.892	0.755	0.882	0.719	0.983	0.601	0.23	0.433	0.0541	0.605
IRS2	1.1	0.678	0.995	0.944	0.806	0.788	0.64	0.801	1.01	0.959	0.905	0.742	0.786	0.961	0.929	0.994	0.987	0.954
IGFBP3	1.17	0.423	0.781	1.03	0.912	0.932	0.71	0.881	0.889	0.612	0.577	0.18	0.363	2.07	0.11	1.76	0.151	0.796
IGFBP4	0.819	0.397	0.984	0.824	0.409	0.745	0.987	0.948	0.789	0.462	1.27	0.447	0.29	1.51	0.344	0.55	0.134	0.0857
IGFBP5	0.629	**0.0421**	0.191	1	0.995	0.599	0.183	0.155	0.571	0.09	2.45	**0.0309**	**0.00606**	0.986	0.969	0.43	0.103	0.208
CDH1	0.961	0.857	0.727	0.833	0.45	0.548	0.661	0.587	1.24	0.602	0.882	0.747	0.549	1.01	0.975	0.843	0.606	0.69
IGFR_sig_Creighton	1.76	**0.0211**	0.171	2.05	**0.00758**	**0.0198**	0.695	0.696	1.48	0.343	1.44	0.341	0.963	1.47	0.324	3.48	**0.012**	0.196
IGFBP5_IGFP4_ratio	0.749	0.176	0.209	1.23	0.427	0.425	0.144	0.156	0.691	0.222	1.54	0.243	0.0937	0.841	0.603	0.961	0.922	0.802
IGF1_ligand_score_Mu	0.534	**0.0143**	0.0939	0.627	0.06	0.114	0.67	0.741	0.576	0.124	0.847	0.593	0.429	0.716	0.446	0.43	0.0544	0.429

* Adjusted for HR

SUPPLEMENTARY REFERENCES

1. Mu, L. *et al.* Favorable outcome associated with an IGF-1 ligand signature in breast cancer. *Breast Cancer Res Tr* **133**, 321–331 (2012).

2. Creighton, C. J. *et al.* Insulin-Like Growth Factor-I Activates Gene Transcription Programs Strongly Associated With Poor Breast Cancer Prognosis. *J Clin Oncol* **26**, 4078–4085 (2008).
