# Supplementary material for: Ganitumab and metformin plus standard neoadjuvant therapy in stage 2/3 breast cancer
Source: NPJ Breast Cancer. 2021 Oct 5;7:131. doi: 10.1038/s41523-021-00337-2 (PMC8492731; doi:10.1038/s41523-021-00337-2)
Supplement: Supplementary file 3 — Reporting Summary [file 41523_2021_337_MOESM3_ESM.pdf]

nature research

Corresponding author(s): Douglas Yee, MD
Masonic Cancer Center, University of Minnesota
420 Delaware Street SE
MMC 806
Minneapolis, MN 55455
Tel: (612) 626-8487
Email: yeexx006@umn.edu

Last updated by author(s): Apr 7, 2021

Reporting Summary

Nature Research wishes to improve the reproducibility of the work that we publish. This form provides structure for consistency and transparency in reporting. For further information on Nature Research policies, see our Editorial Policies and the Editorial Policy Checklist.

Statistics

For all statistical analyses, confirm that the following items are present in the figure legend, table legend, main text, or Methods section.

n/a	Confirmed	
☐	☒	The exact sample size (*n*) for each experimental group/condition, given as a discrete number and unit of measurement
☐	☒	A statement on whether measurements were taken from distinct samples or whether the same sample was measured repeatedly
☐	☒	The statistical test(s) used AND whether they are one- or two-sided<br>*Only common tests should be described solely by name; describe more complex techniques in the Methods section.*
☐	☒	A description of all covariates tested
☐	☒	A description of any assumptions or corrections, such as tests of normality and adjustment for multiple comparisons
☐	☒	A full description of the statistical parameters including central tendency (e.g. means) or other basic estimates (e.g. regression coefficient) AND variation (e.g. standard deviation) or associated estimates of uncertainty (e.g. confidence intervals)
☐	☒	For null hypothesis testing, the test statistic (e.g. *F*, *t*, *r*) with confidence intervals, effect sizes, degrees of freedom and *P* value noted<br>*Give P values as exact values whenever suitable.*
☐	☒	For Bayesian analysis, information on the choice of priors and Markov chain Monte Carlo settings
☒	☐	For hierarchical and complex designs, identification of the appropriate level for tests and full reporting of outcomes
☒	☐	Estimates of effect sizes (e.g. Cohen's *d*, Pearson's *r*), indicating how they were calculated

Our web collection on statistics for biologists contains articles on many of the points above.

Software and code

Policy information about availability of computer code

Data collection	Clinical data are entered at each I-SPY 2 site via case report forms into a Salesforce database and reviewed by the trial sponsor's, Quantumleap Healthcare, data management team. Gene expression microarray intensity values are derived from Agilent Feature Extraction files. DataPrint, Agendia's proprietary software for extracting data from Agilent Feature Extraction files, performs log2 transformation on the gMeanSignal. The 75th quantile is then subtracted from the entire array, and a fixed value of 9.5 added.
Data analysis	The randomization engine and Bayesian analytic software used in efficacy analysis are used under license from Berry Consultants, LLC; requests for code should be directed to don@berryconsultants.com. Biomarker data analysis was performed using R version 3.4.3; code available upon request from ispyadmin@ucsf.edu.

For manuscripts utilizing custom algorithms or software that are central to the research but not yet described in published literature, software must be made available to editors and reviewers. We strongly encourage code deposition in a community repository (e.g. GitHub). See the Nature Research guidelines for submitting code & software for further information.

Data

Policy information about availability of data

All manuscripts must include a data availability statement. This statement should provide the following information, where applicable:

- Accession codes, unique identifiers, or web links for publicly available datasets
- A list of figures that have associated raw data
- A description of any restrictions on data availability

Clinical dataset that support Figure 1 and Supplemental Figures 1 and 2 are available upon request by email to ispyadmin@ucsf.edu.
Biomarker data supporting Figure 2 and 3 has been provided as a supplementary file.

Field-specific reporting

Please select the one below that is the best fit for your research. If you are not sure, read the appropriate sections before making your selection.

☒ Life sciences ☐ Behavioural & social sciences ☐ Ecological, evolutionary & environmental sciences

For a reference copy of the document with all sections, see nature.com/documents/nr-reporting-summary-flat.pdf

Life sciences study design

All studies must disclose on these points even when the disclosure is negative.

Sample size	I-SPY2 is adaptively randomized, so number of participants in arm is not fixed. Enrollment to arms is halted when: 1) Agents 'graduate' from I-SPY2 by reaching, in any of 10 clinically relevant signatures, a predefined efficacy threshold of 85% probability of success in a signature-specific, hypothetical 300-patient, 1:1 confirmatory phase 3 trial. (2) Agents may be dropped for futility if the predicted probability <10% for all signatures or the maximum enrollment threshold has been reached for that arm. (3) Agents may exit due to reaching maximum accrual if it has predicted probability of success >10% and <85% (4) or be dropped for safety as recommended by an independent DSMB.
Data exclusions	No data exclusion
Replication	Not applicable - Clinical trial
Randomization	Biomarker assessments at screening are used to assess eligibility and classify patients into one of eight subtypes based on hormone receptor (HR), HER2-receptor and Mammaprint (high vs. ultra-high) status. Adaptive randomization in I-SPY2 preferentially assigns patients to agents according to actively updated Bayesian posterior probabilities of pCR rate within each subtype; 20% of patients are randomized to control.
Blinding	Investigators are not blinded to randomization results, but are blinded to efficacy analysis findings until announcement that experimental regimens have exited the trial.

Reporting for specific materials, systems and methods

We require information from authors about some types of materials, experimental systems and methods used in many studies. Here, indicate whether each material, system or method listed is relevant to your study. If you are not sure if a list item applies to your research, read the appropriate section before selecting a response.

Materials & experimental systems

n/a	Involved in the study	
☒	☐	Antibodies
☒	☐	Eukaryotic cell lines
☒	☐	Palaeontology and archaeology
☒	☐	Animals and other organisms
☐	☒	Human research participants
☐	☒	Clinical data
☒	☐	Dual use research of concern

Methods

n/a	Involved in the study	
☒	☐	ChIP-seq
☒	☐	Flow cytometry
☒	☐	MRI-based neuroimaging

Human research participants

Policy information about studies involving human research participants

Population characteristics	Between July 2012 and February 2015, 106 patients with HER2-negative tumors received paclitaxel/ganitumab/metformin (PGM) while 128 contemporary control patients (derived from the start of the trial in March 2010 through deactivation of the PGM arm in February 2015) received paclitaxel alone (Figure 1). In the PGM arm, 14 patients did not receive assigned therapy and in the standard of care control arm, 9 patients did not receive allocated therapy and are not included in the

	analysis. Patients with HER2-positive tumors were not included in the PGM arm due to the lack of safety data for ganitumab in combination with trastuzumab. All patients received AC after completing the regimen. Baseline characteristics were similar between both arms, although there were slightly more participants with MammaPrint Hi2 status (56% versus 45%) in the experimental arm (Table 1).
Recruitment	Recruited by individual investigators from local patient populations at their clinical site
Ethics oversight	Institutional Review Boards at each clinical site

Note that full information on the approval of the study protocol must also be provided in the manuscript.

Clinical data

Policy information about clinical studies

All manuscripts should comply with the ICMJE guidelines for publication of clinical research and a completed CONSORT checklist must be included with all submissions.

Clinical trial registration	NCT01042379
Study protocol	Upon request
Data collection	July 2012 through February 2015. Clinical (and lab test) data collected at each I-SPY 2 site; and expression data collected at Agendia.
Outcomes	The primary endpoint is pathological complete response (pCR) after completion of all chemotherapy, defined as the absence of invasive tumor in breast and regional nodes (ypT0/is and ypN0). Assessed using the Residual Cancer Burden (RCB) method at the time of surgery. Secondary endpoints include RCB, a continuous measure that quantitates the extent of residual disease (where RCB of 0 is a pCR), and event-free survival (EFS). Patients are followed for long term outcomes (local recurrence, distant recurrence or death); and follow-up data is collected and updated yearly. EFS is calculated as the time between treatment consent and first local or distant recurrence or death; and patients without event were censored at time to last follow-up.
